# Supplementary material for: Atomic force microscopy-single-molecule force spectroscopy unveils GPCR cell surface architecture
Source: Commun Biol. 2022 Mar 10;5:221. doi: 10.1038/s42003-022-03162-w (PMC8913689; doi:10.1038/s42003-022-03162-w)
Supplement: Supplementary file 2 — Supplementary Information [file 42003_2022_3162_MOESM2_ESM.pdf]

Supplementary Figures and Table

Supplementary Figure 1

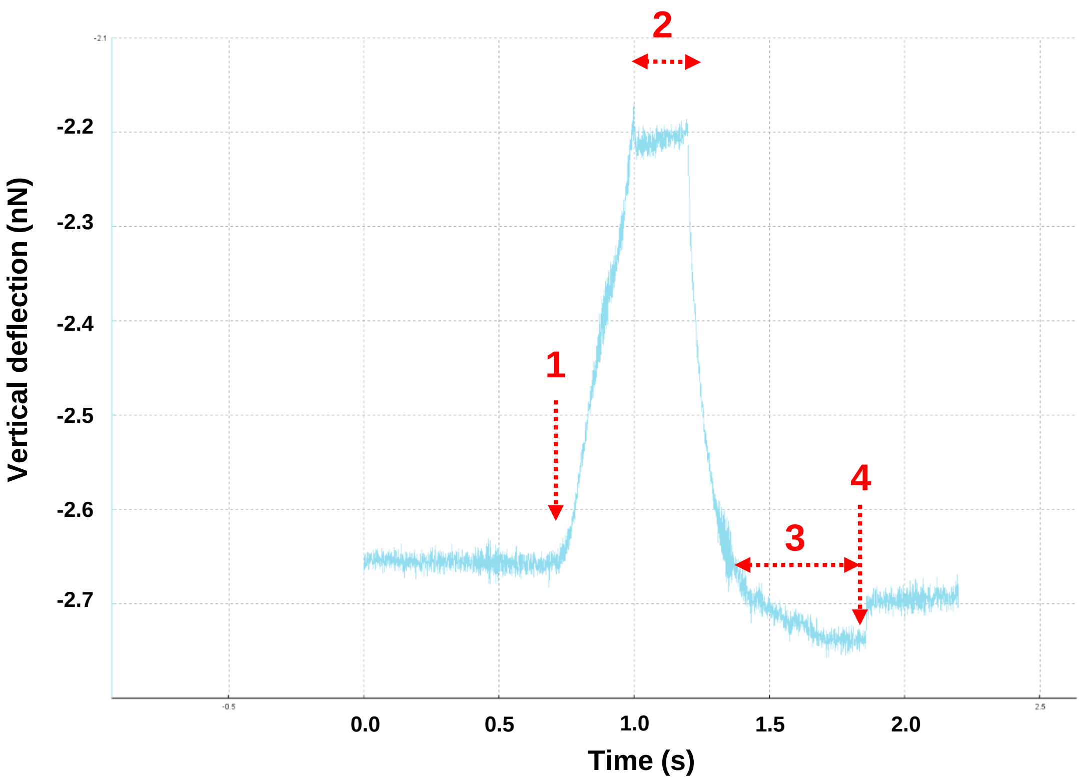

Supplementary Figure 1. Force-time curve recorded by AFM-SMFS on one approach-retraction cycle. Representative force-time curve of one approach-retraction cycle showing **1)** the approach of the tip to the cell surface, **2)** the 200 ms pause of the tip at the cell surface to optimize the probability of a single interaction between the HA antibody and the HA-GPCR, **3)** the retraction of the tip from the surface with the unfolding events in case of the existence of adhesive forces between the tip and the surface and **4)** the rupture of the tip / surface interaction with the return to the baseline.

Supplementary Figure 2

Dague et al

a **Excluded curves**

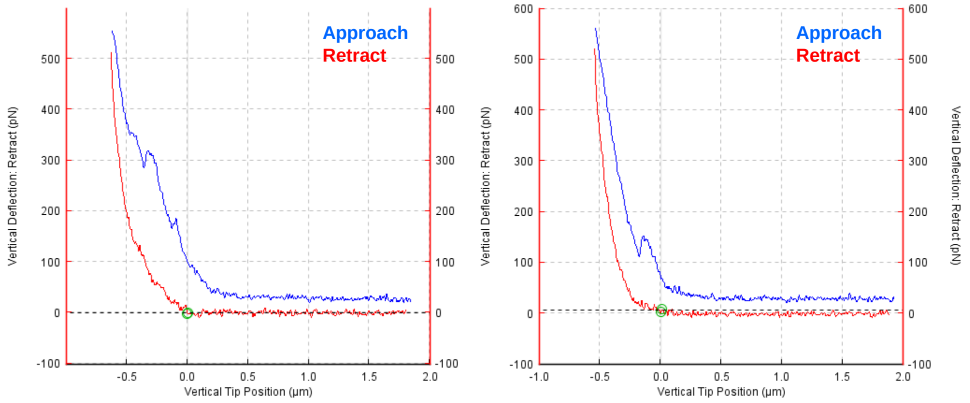

b **Non-adhesive (NA) curves**

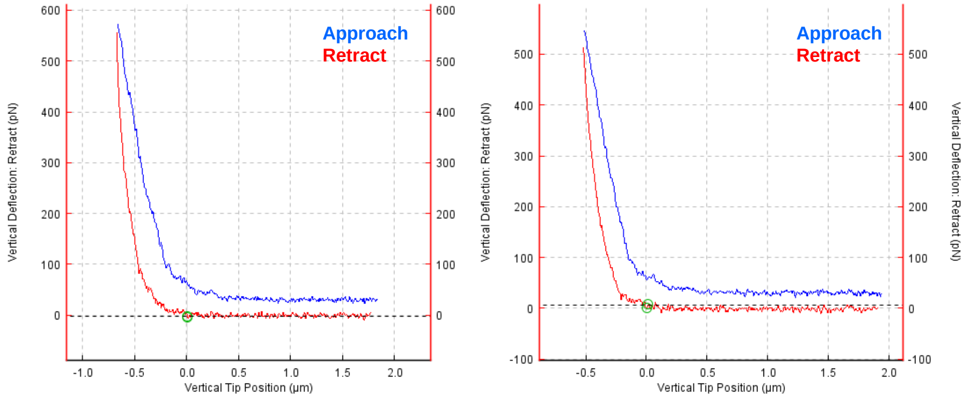

c **Adhesive curves**

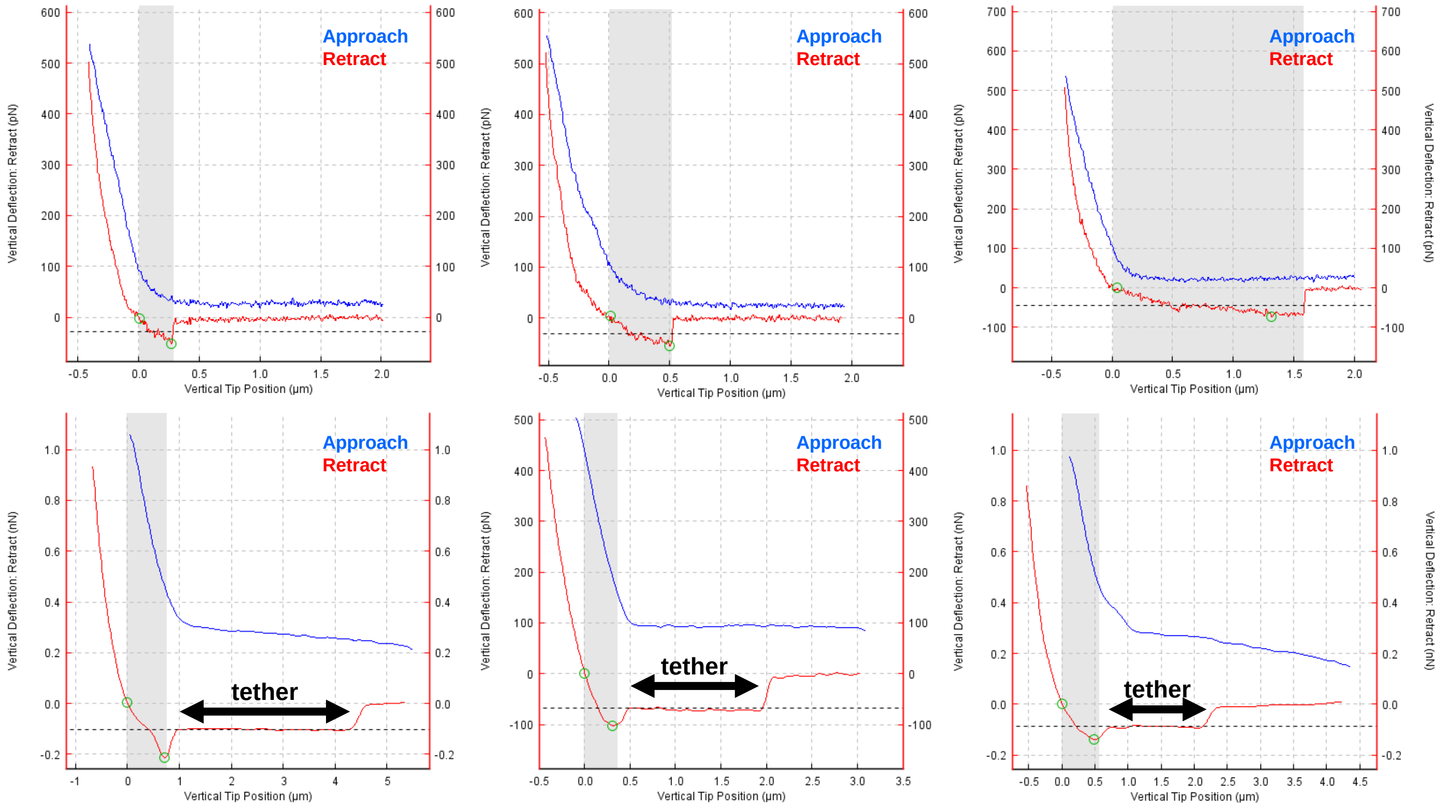

Supplementary Figure 2. Force-distance curve selection criteria used for SMFS-based GPCR unfolding at the living WTT-CHO cell surface. Representative FD curves showing examples of excluded curves **(a)**, non-adhesive (NA) **(b)** and adhesive **(c)** curves and the measurement of GPCR unfolding lengths (**c**, gray area) relying on the distance between the contact point and the point of lowest force on the retraction force curve without taking into account the membrane tethers (left-right black arrow).

Supplementary Figure 3

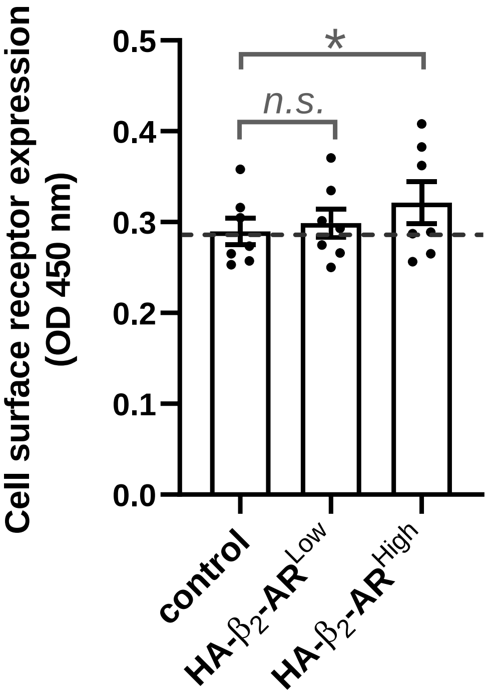

Supplementary Figure 3. HA-β_2-AR expression at the WTT-CHO cell surface. WTT-CHO cells were transfected with 0.1 or 1 µg of HA-β_2-AR-encoding vector (HA-β_2-AR^{Low} and HA-β_2-AR^{High} respectively) or the empty vector (control). Cell surface expression of HA-β_2-AR was quantified by ELISA using an anti-HA antibody. Data represent the mean ± s.e.m. of seven independent experiments. The statistical significance of differences between HA-β_2-AR-expressing cells and control cells was assessed using one-way ANOVA followed by Sidak's multiple comparisons tests (* $p < 0.05$; n.s., not statistically significant).

Supplementary Figure 4

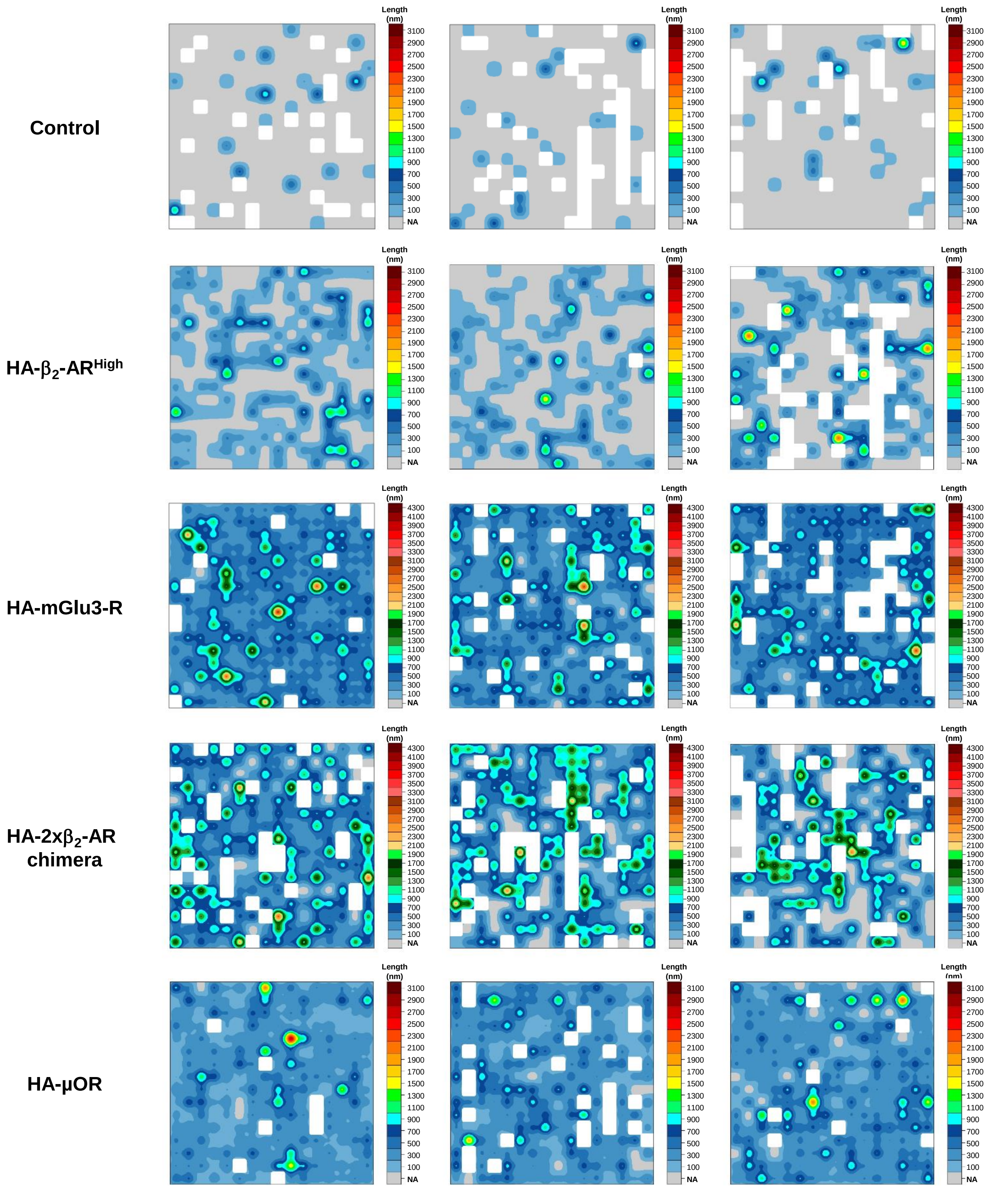

Supplementary Figure 4. Representative spatial maps of HA-GPCR unfoldings. The different spatial maps were obtained after analyzing SMFS experiments conducted on WTT-CHO cells transiently transfected with the empty vector as a negative control (control) or vectors encoding the different HA-GPCRs. Spatial maps are representative of different cells and/or different experiments.

Supplementary Figure 4 (continued)

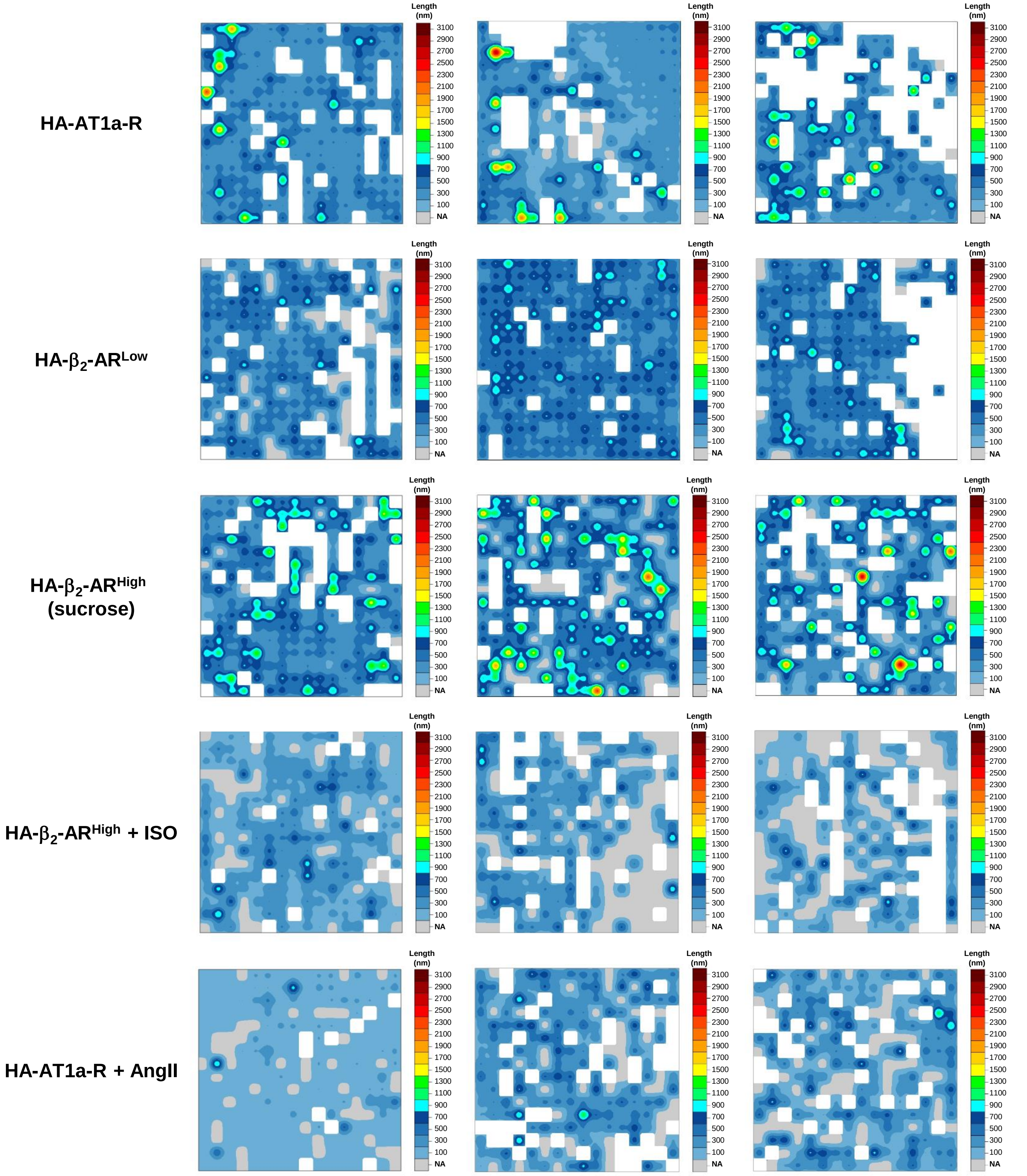

Supplementary Figure 4. Representative spatial maps of HA-GPCR unfoldings. The different spatial maps were obtained after analyzing SMFS experiments conducted on WTT-CHO cells transiently transfected with the empty vector as a negative control (control) or vectors encoding the different HA-GPCRs. Spatial maps are representative of different cells and/or different experiments.

Supplementary Figure 5

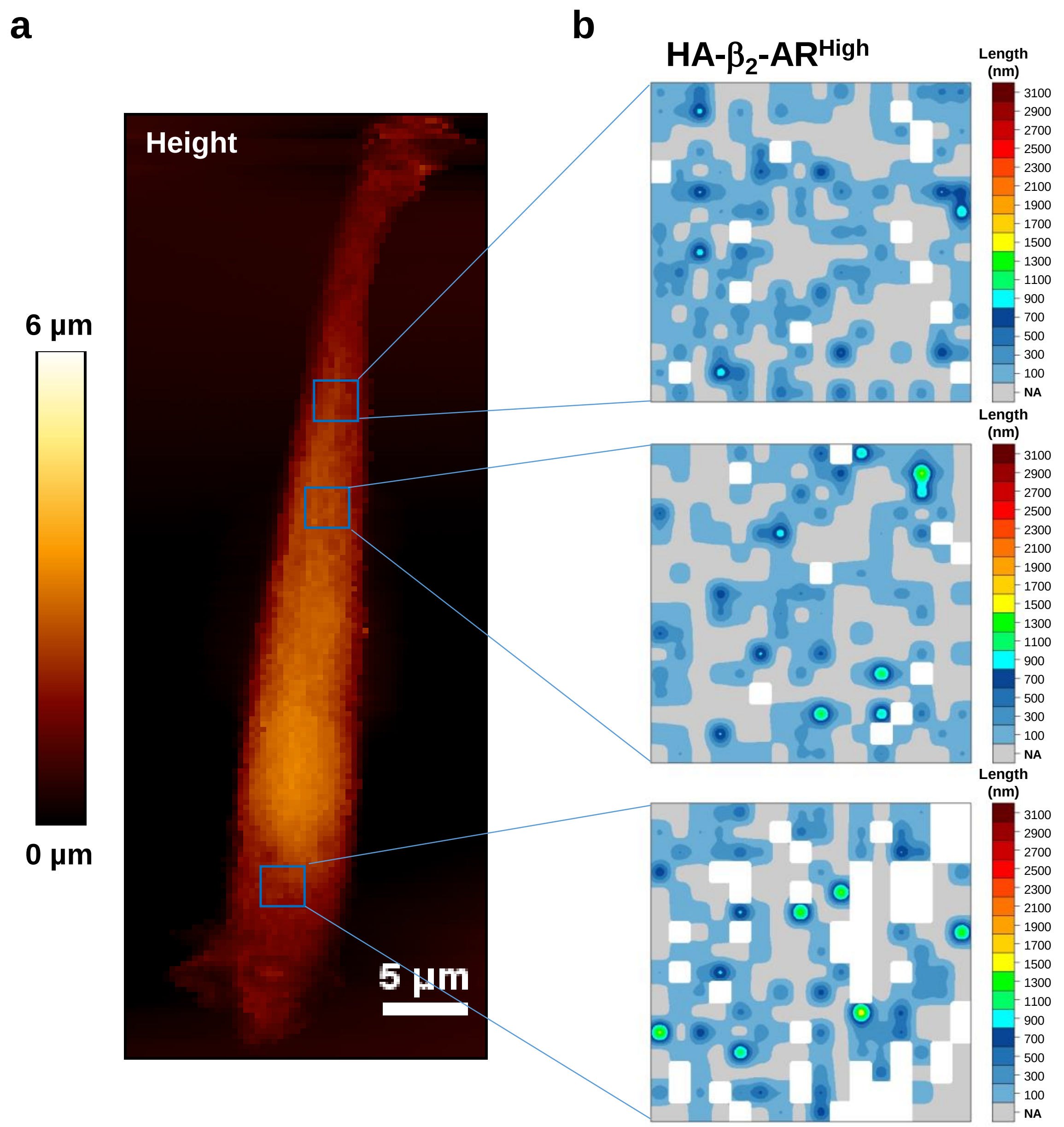

Supplementary Figure 5. Homogenous HA-β_2-AR spatial organization at the surface of WTT-CHO cells. WTT-CHO cells were transiently transfected with 1 µg of HA-β_2-AR-encoding vector (HA-β_2-AR^{High}) and SMFS experiments were conducted on three different areas of one cell. The representative AFM height image (**a**) and spatial maps of the unfolding lengths/non-adhesive events of three different areas of one cell (**b**) are shown.

Supplementary Figure 6

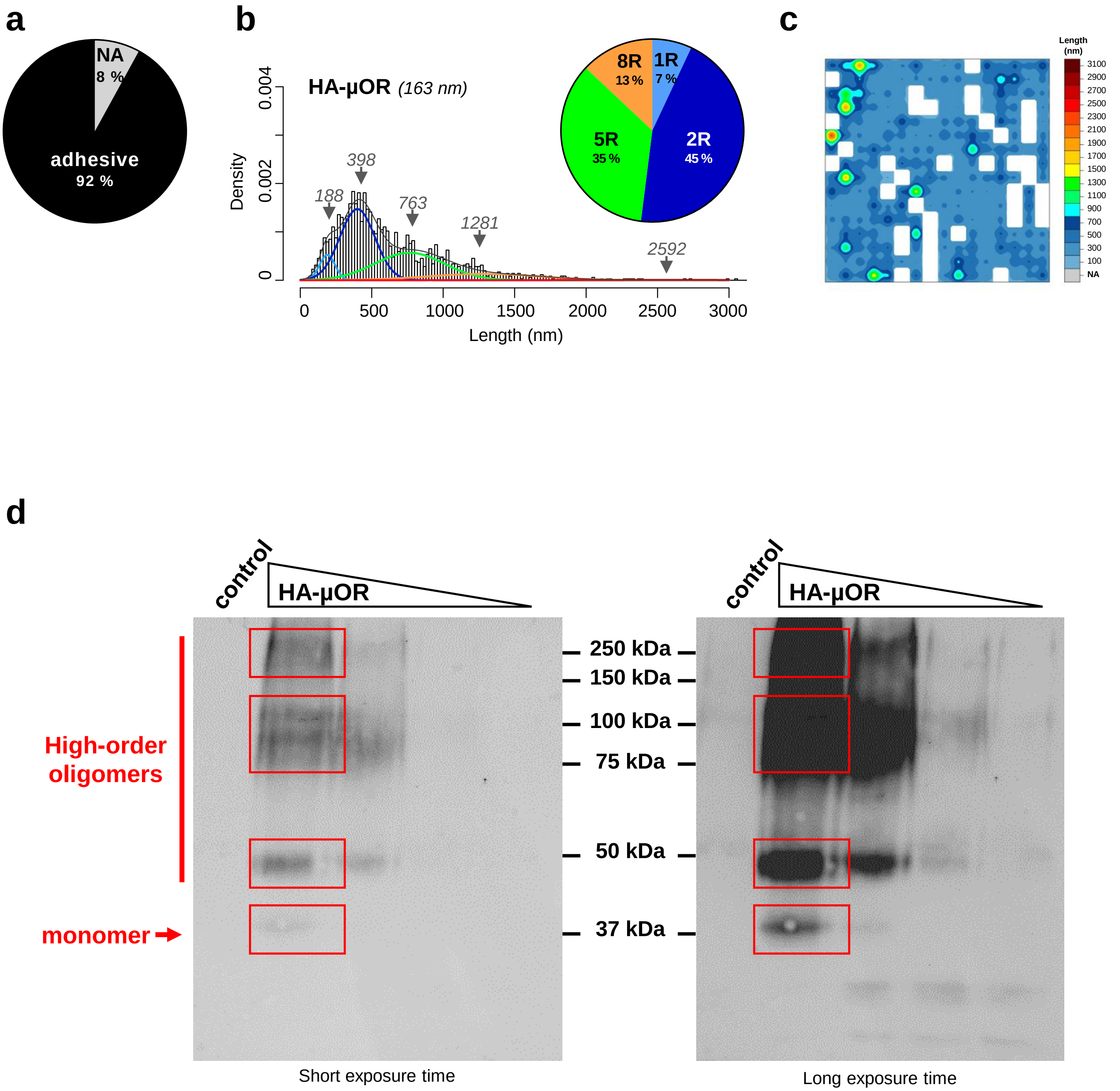

Supplementary Figure 6. Oligomerization of HA-µOR assessed by AFM-SMFS or Western-blotting in WTT-CHO cells. SMFS experiments were conducted on WTT-CHO cells transiently transfected with HA-µOR-encoding vector. The proportions of adhesive and non-adhesive (NA) events are shown in the pie chart (**a**). Unfolding distances were analyzed by fitting with a Gaussian mixture according to the BIC-based method. Each Gaussian population was assigned to receptor oligomeric state (*1 receptor/monomer=1R; 2 receptors/dimer=2R; 3 receptors/trimer=3R…*) based on its theoretical length, and Gaussian weight is presented in the pie chart (**b**). The 256 unfolding distances resulting from one representative cell area (3x3 µm^2) were depicted as a spatial map (**c**). At least nine cells from three independent experiments were analyzed. (**d**) Cell lysates from WTT-CHO cells transiently transfected with the empty vector (control) or with decreasing amounts of HA-µOR-encoding vector were subjected to immunoprecipitation and then analyzed by SDS gel electrophoresis and western blotting using an anti-HA antibody. Short and long exposure times were shown.

Supplementary Figure 7

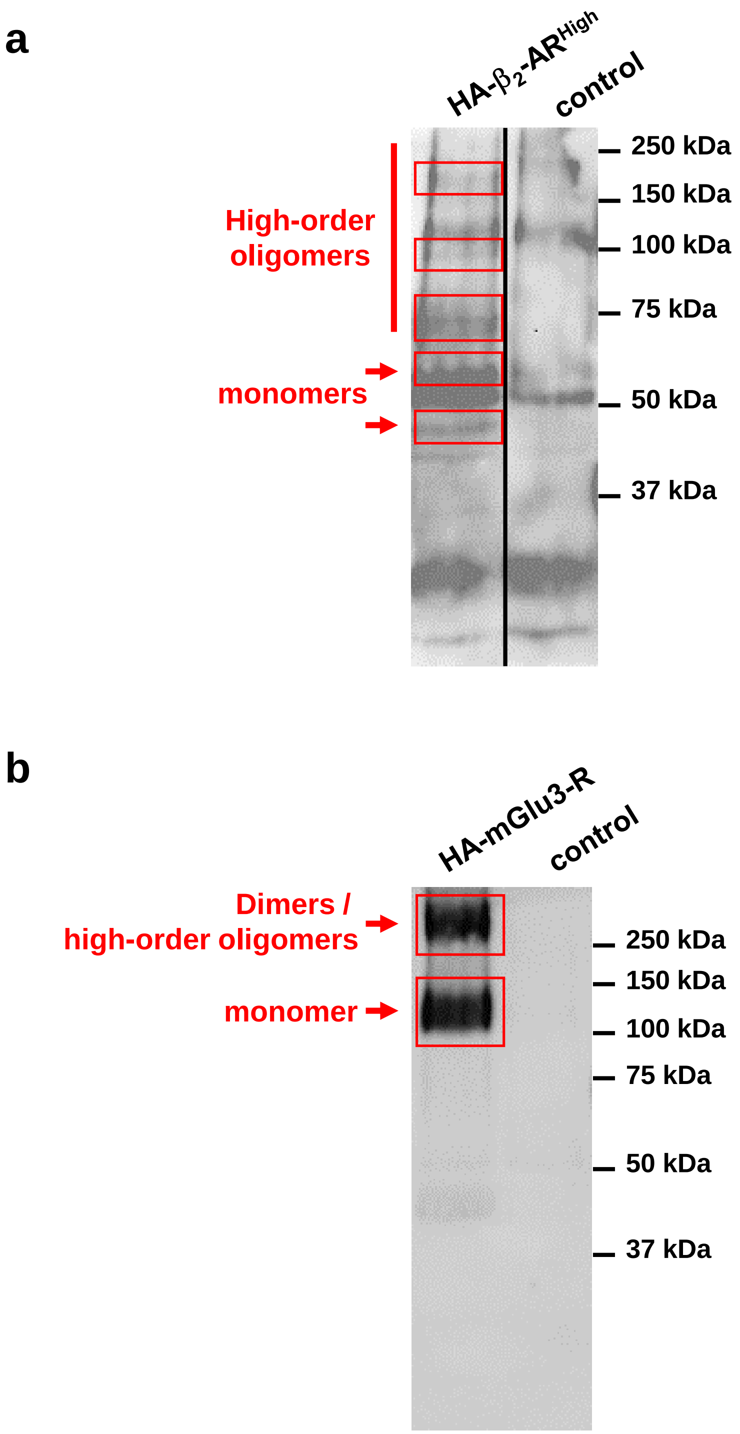

Supplementary Figure 7. Oligomerization of HA-β_2-AR or HA-mGlu3-R assessed by Western-blotting in WTT-CHO cells. Cell lysates from WTT-CHO cells transiently transfected with the empty vector (control) or with vector encoding HA-β_2-AR (**a**) or HA-mGlu3-R (**b**) were subjected to immunoprecipitation using an anti-HA antibody and then analyzed by SDS gel electrophoresis and Western-blotting using the anti-HA antibody.

Supplementary Figure 8

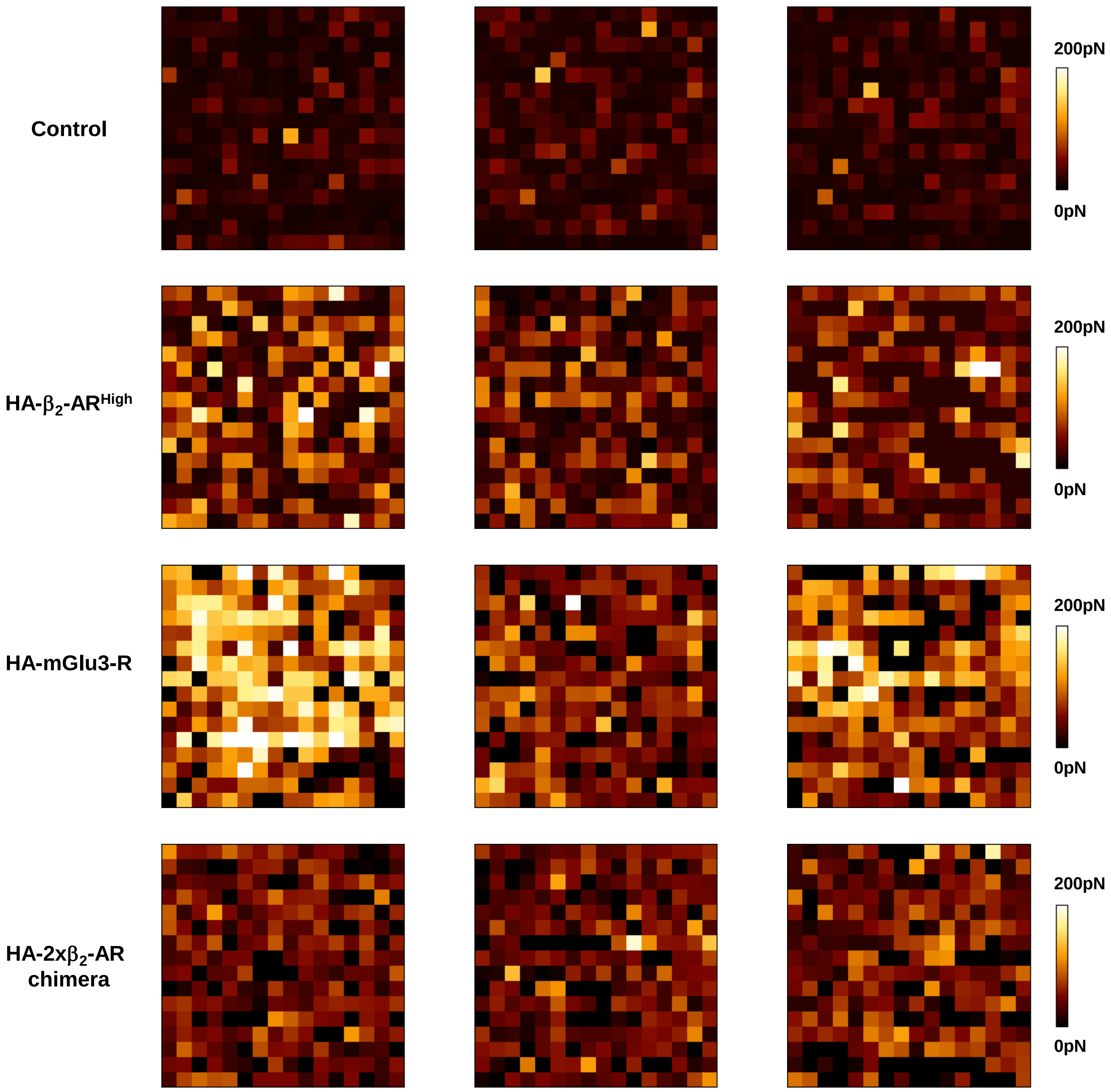

Supplementary Figure 8. Representative force rupture maps in WTT-CHO cells expressing or not HA-GPCRs. SMFS experiments were conducted on WTT-CHO cells transiently transfected with the empty vector (control) or with vector encoding for HA-β_2-AR, HA-mGlu3-R or HA-2xβ_2-AR chimera and recorded adhesion forces were depicted as a spatial map. Spatial maps are representative of different cells and/or different experiments.

Supplementary Figure 9

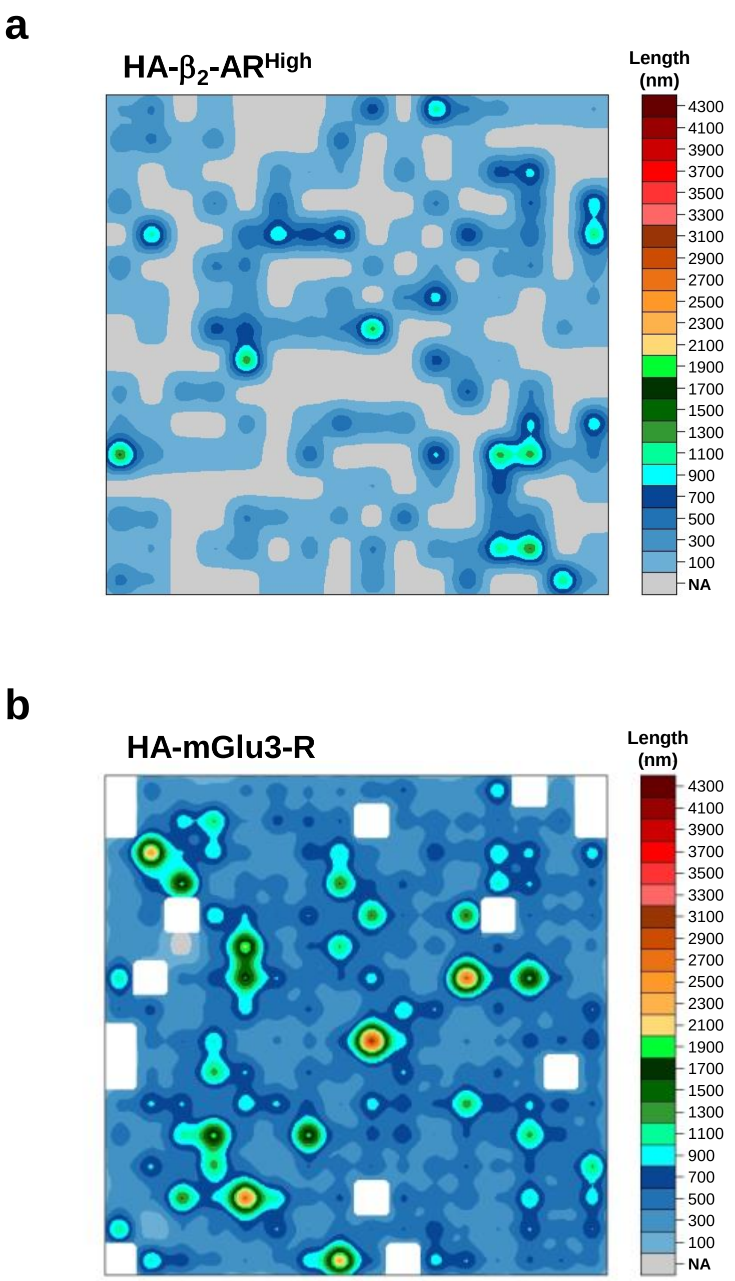

Supplementary Figure 9. Comparison of the spatial organization of HA-β_2-AR and HA-mGlu3-R unfoldings. SMFS experiments were conducted at the basal state on WTT-CHO cells transiently transfected with 1 µg of vector encoding HA-β_2-AR (HA-β_2-AR^{High}) (**a**) or HA-mGlu3-R **(b)**. The 256 unfolding distances resulting from one representative cell area (3x3 µm^2) (**data from Fig. 4e, f**) are depicted as a spatial map but using the HA-mGlu3-R length scale to allow comparative analysis.

Supplementary Figure 10

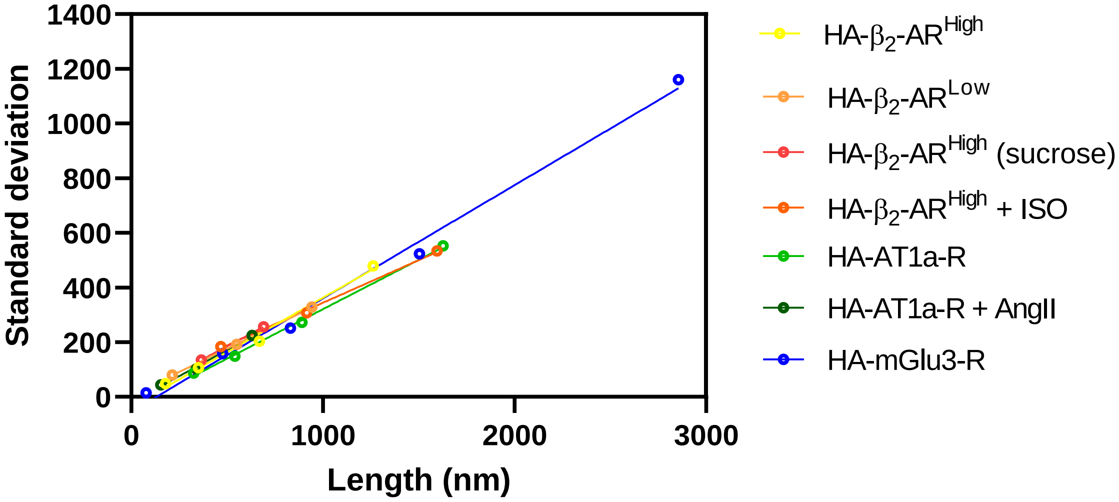

	HA-β_2-AR^{High}	HA-β_2-AR^{Low}	HA-β_2-AR^{High} (sucrose)	HA-β_2-AR^{High} + ISO	HA-AT1a-R	HA-AT1a-R + AngII	HA-mGlu3-R
r^2	0,9907	0,9998	1	0,9972	0,9981	0,9939	0,9925
P value	0,0047	0,0100	perfect line	0,0335	0,0009	0,0497	0,0003

Supplementary Figure 10. Validity of the GPCR-unfolding length assessment. The correlation between the standard deviation and the mean of each Gaussian population was assessed for each AFM-SMFS experimental condition using the Spearman test.

Supplementary Figure 11

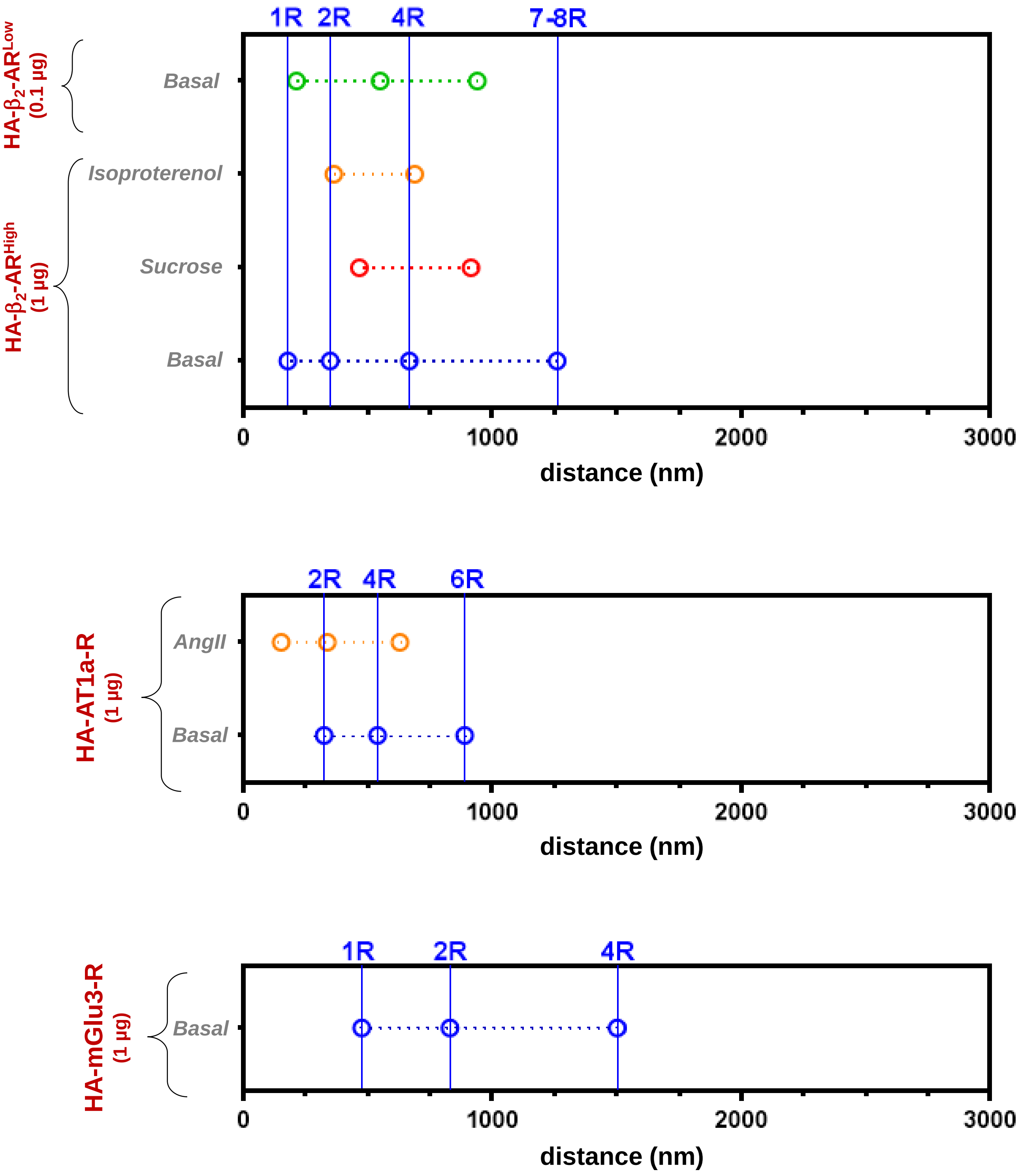

Supplementary Figure 11. Schematics of the GPCR oligomeric states assessed by AFM-SMFS. For each GPCR tested (HA-β_2-AR, HA-AT1a-R, HA-mGlu3-R), the mean of each Gaussian population (**obtained in Fig. 4, 5 and 6**) was plotted and compared to those of the basal state depicted in cells transfected with 1 µg of GPCR-encoding vector (plain blue lines). Each Gaussian population was assigned to a GPCR oligomeric state (*1 receptor/monomer=1R; 2 receptors/dimer=2R; 4 receptors/tetramer=4R…*) based on their theoretical length using their amino-acid sequence.

Supplementary Table 1

na = not available				Gaussian 1			Gaussian 2			Gaussian 3			Gaussian 4			Gaussian 5		
**Experimental condition**	**Analysis Method**	**unfolding number**	**Gaussian number (BIC)**	**Mean**	**Standard deviation**	**Weight**	**Mean**	**Standard deviation**	**Weight**	**Mean**	**Standard deviation**	**Weight**	**Mean**	**Standard deviation**	**Weight**	**Mean**	**Standard deviation**	**Weight**
**HA-β_2-AR^{High}**	**NormalMixEm**	**1759**	**4**	179,29	48,37421	0,1608713	350,6333	106,28181	0,3917083	669,029	204,00567	0,3173776	1262,3499	478,64009	0,1300428	na	na	na
**HA-β_2-AR^{Low}**	**NormalMixEm**	**1550**	**3**	214,6334	80,25151	0,0892309	551,0868	191,38186	0,808167	942,7587	328,0479	0,10260216	na	na	na	na	na	na
**HA-β_2-AR^{High} + ISO**	**NormalMixEm**	**1260**	**2**	365,8863	135,1998	0,6997981	690,9261	256,2226	0,3002019	na	na	na	na	na	na	na	na	na
**HA-β_2-AR^{High} (sucrose)**	**NormalMixEm**	**1565**	**3**	467,1702	184,2742	0,7072008	916,5263	307,2004	0,2465224	1596,0131	533,9793	0,04627681	na	na	na	na	na	na
**HA-AT1a-R**	**NormalMixEm**	**1634**	**4**	326,4678	86,86959	0,4012591	540,7161	148,53622	0,4214503	891,5909	272,2241	0,14080172	1628,3042	552,36227	0,03648888	na	na	na
**HA-AT1a-R + AngII**	**NormalMixEm**	**1102**	**3**	153,9476	43,98549	0,1437738	338,9259	101,60873	0,652144	630,9669	225,1216	0,2040822	na	na	na	na	na	na
**HA-mGlu3-R**	**NormalMixEm**	**1926**	**5**	77,97623	15,15942	0,044522	477,13141	158,36232	0,3573475	831,37724	251,75769	0,36267622	1504,55235	523,25683	0,19329861	2855,13195	1159,90962	0,04215564
**HA-2xβ_2-AR chimera**	**NormalMixEm**	**1821**	**5**	63,48552014	11,63369834	0,0303672	349,8625757	110,1937699	0,2276649	610,4944977	171,4950027	0,270103727	1004,266136	276,4174555	0,329418312	1611,436051	533,768908	0,142445919
**HA-μOR**	**NormalMixEm**	**1771**	**5**	188,139	54,1294	0,070837	398,6502	122,3993	0,450849	763,3508	242,4233	0,34407	1281,645	387,6354	0,126423	2592,534	635,3475	0,007821

Supplementary Table 1. Summary of mathematical analyses of Gaussian distribution of the receptor unfolding distances. Unfolding distances measured by AFM-SMFS were pooled for each experimental condition and analyzed by fitting with a Gaussian mixture using NormalMixEm according to the BIC-based method. The table indicates the characteristics of the Gaussian distributions (number, mean, standard deviation, weight).
