# Supplementary material for: Atomic force microscopy-single-molecule force spectroscopy unveils GPCR cell surface architecture
Source: Commun Biol. 2022 Mar 10;5:221. doi: 10.1038/s42003-022-03162-w (PMC8913689; doi:10.1038/s42003-022-03162-w)
Supplement: Supplementary file 3 — Reporting Summary [file 42003_2022_3162_MOESM3_ESM.pdf]

nature portfolio

COMMSBIO-20-3256A
Corresponding author(s): DAGUE Étienne
GALES Céline

Last updated by author(s): Jan 25, 2022

Reporting Summary

Nature Portfolio wishes to improve the reproducibility of the work that we publish. This form provides structure for consistency and transparency in reporting. For further information on Nature Portfolio policies, see our Editorial Policies and the Editorial Policy Checklist.

Statistics

For all statistical analyses, confirm that the following items are present in the figure legend, table legend, main text, or Methods section.

n/a	Confirmed	
☐	☒	The exact sample size (*n*) for each experimental group/condition, given as a discrete number and unit of measurement
☐	☒	A statement on whether measurements were taken from distinct samples or whether the same sample was measured repeatedly
☐	☒	The statistical test(s) used AND whether they are one- or two-sided<br>*Only common tests should be described solely by name; describe more complex techniques in the Methods section.*
☒	☐	A description of all covariates tested
☐	☒	A description of any assumptions or corrections, such as tests of normality and adjustment for multiple comparisons
☐	☒	A full description of the statistical parameters including central tendency (e.g. means) or other basic estimates (e.g. regression coefficient) AND variation (e.g. standard deviation) or associated estimates of uncertainty (e.g. confidence intervals)
☒	☐	For null hypothesis testing, the test statistic (e.g. *F*, *t*, *r*) with confidence intervals, effect sizes, degrees of freedom and *P* value noted<br>*Give P values as exact values whenever suitable.*
☒	☐	For Bayesian analysis, information on the choice of priors and Markov chain Monte Carlo settings
☒	☐	For hierarchical and complex designs, identification of the appropriate level for tests and full reporting of outcomes
☒	☐	Estimates of effect sizes (e.g. Cohen's *d*, Pearson's *r*), indicating how they were calculated

Our web collection on statistics for biologists contains articles on many of the points above.

Software and code

Policy information about availability of computer code

Data collection	AFM force maps were collected using JPKSPM data processing software
Data analysis	Gaussian Mixture analysis and Spatial analysis were conducted in R studio v1.2.5033, R v3.6.3 using mixtools v1.2.0

For manuscripts utilizing custom algorithms or software that are central to the research but not yet described in published literature, software must be made available to editors and reviewers. We strongly encourage code deposition in a community repository (e.g. GitHub). See the Nature Portfolio guidelines for submitting code & software for further information.

Data

Policy information about availability of data

All manuscripts must include a data availability statement. This statement should provide the following information, where applicable:

- Accession codes, unique identifiers, or web links for publicly available datasets
- A description of any restrictions on data availability
- For clinical datasets or third party data, please ensure that the statement adheres to our policy

The dataset generated and/or analyzed during the current study are available from the corresponding author on request

Field-specific reporting

Please select the one below that is the best fit for your research. If you are not sure, read the appropriate sections before making your selection.

☒ Life sciences ☐ Behavioural & social sciences ☐ Ecological, evolutionary & environmental sciences

For a reference copy of the document with all sections, see nature.com/documents/nr-reporting-summary-flat.pdf

Life sciences study design

All studies must disclose on these points even when the disclosure is negative.

Sample size	*For the Gaussian analysis of SMFS unfoldings, since no data allowing calculation of sample size were available, a similar number of SMFS-unfoldings were collected for each experimental condition (around 1500 unfoldings curves / experimental condition)<br>*For the classical ELISA and quantification of cAMP production, sample size was based on our previous experience
Data exclusions	For the SMFS-unfolding data, exclusion criteria of the unfolding curves have been described in the manuscript
Replication	For each condition, experiments were repeated at least 3 times as indicated in the figure legends
Randomization	not applicable
Blinding	For the evaluation of the SMFS-unfolding distances, the manual analysis was performed by at least 2 experimenters blinded to the experimental conditions.

Reporting for specific materials, systems and methods

We require information from authors about some types of materials, experimental systems and methods used in many studies. Here, indicate whether each material, system or method listed is relevant to your study. If you are not sure if a list item applies to your research, read the appropriate section before selecting a response.

Materials & experimental systems

n/a	Involved in the study	
☐	☒	Antibodies
☐	☒	Eukaryotic cell lines
☒	☐	Palaeontology and archaeology
☒	☐	Animals and other organisms
☒	☐	Human research participants
☒	☐	Clinical data
☒	☐	Dual use research of concern

Methods

n/a	Involved in the study	
☒	☐	ChIP-seq
☒	☐	Flow cytometry
☒	☐	MRI-based neuroimaging

Antibodies

Antibodies used	* Anti-HA antibody used for the SMFS experiments has described previously (Formosa C. et al, J. Mol. Recognit. 2015; 28:1-9) : LifeProTein, Ref:LT0422, HA.C5 clone monoclonal antibody<br>*anti-HA used for the cell-surface ELISA experiments: BioLegend, 16B12 Clone<br>*HRP-labeled secondary antibody used for the cell-surface ELISA experiments: Sigma, St. Louis, MO, USA, Ref : A4416<br>*HRP-labeled secondary antibody used for the Western-blot experiments: anti-mouse Horse Radish Peroxidase (HRP)-conjugated secondary antibodies (TrueBlot, eB144Clone, Rockland)
Validation	Validation of anti-HA antibodies used for SMFS and ELISA experiments were performed by comparing cells expressing or not (negative control) the HA-GPCR

Eukaryotic cell lines

Policy information about cell lines

Cell line source(s)	WTT-CHO cells used in this study were provided by Dr M.P. Rols and already described in : Rols, M.P. & Teissie, J. Experimental evidence for the involvement of the cytoskeleton in mammalian cell electropermeabilization. Biochim Biophys Acta 1111, 45-50 (1992).
Authentication	the WTT-CHO cell line was not authenticated
Mycoplasma contamination	the cell line was not tested for mycoplasma contamination

March 2021

Commonly misidentified lines
(See ICLAC register)

Name any commonly misidentified cell lines used in the study and provide a rationale for their use.
